# Dried Fruits, Nuts, and Cancer Risk and Survival: A Review of the Evidence and Future Research Directions

**DOI:** 10.3390/nu15061443

**Published:** 2023-03-16

**Authors:** Bradley W. Bolling, Dagfinn Aune, Hwayoung Noh, Kristina S. Petersen, Heinz Freisling

**Affiliations:** 1 Department of Food Science, University of Wisconsin-Madison, 1605 Linden Dr., Madison, WI 53706, USA; 2 Department of Epidemiology and Biostatistics, School of Public Health, Imperial College London, London W2 1PG, UK; 3 Department of Nutrition, Oslo New University College, Lovisenberggata 13, 0456 Oslo, Norway; 4 Department of Endocrinology, Morbid Obesity and Preventive Medicine, Oslo University Hospital Ullevål, 0424 Oslo, Norway; 5 Department of Cancer Prevention and Environment, INSERM U1296, Léon Bérard Cancer Center, 28 Rue Laennec, 69008 Lyon, France; 6 Nutrition and Metabolism Branch, International Agency for Research on Cancer (IARC-WHO), 25 Avenue Tony Garnier, CS 90627, CEDEX 07, 69366 Lyon, France; 7 Department of Nutritional Sciences, Texas Tech University, 508 Human Sciences Building, Lubbock, TX 79409, USA

**Keywords:** dried fruits, tree nuts, peanuts, cancer, cancer survivors, mortality, review

## Abstract

Dried fruits and nuts contain high amounts of nutrients and phytochemicals—all of which may have anticarcinogenic, anti-inflammatory, and antioxidant properties. This narrative review summarizes the evidence for dried fruits and nuts and cancer incidence, mortality, and survival and their potential anticancer properties. The evidence for dried fruits in cancer outcomes is limited, but existing studies have suggested an inverse relationship between total dried fruit consumption and cancer risk. A higher consumption of nuts has been associated with a reduced risk of several site-specific cancers in prospective cohort studies, including cancers of the colon, lung, and pancreas, with relative risks per 5 g/day increment equal to 0.75 (95% CI 0.60, 0.94), 0.97 (95% CI 0.95, 0.98), and 0.94 (95% CI 0.89, 0.99), respectively. A daily intake of total nuts of 28 g/day has also been associated with a 21% reduction in the rate of cancer mortality. There is also some evidence that frequent nut consumption is associated with improved survival outcomes among patients with colorectal, breast, and prostate cancer; however, further studies are needed. Future research directions include the investigation of additional cancer types, including rare types of cancer. For cancer prognosis, additional studies with pre- and postdiagnosis dietary assessment are warranted.

## 1. Introduction

Globally, an estimated 19.3 million new cancer cases and almost 10.0 million cancer deaths occurred in 2020 [1]. In the next two decades, the cancer burden is projected to increase by 47% to 28.4 million new cancer cases in 2040 [1]. This estimate is solely based on the growth and aging of the population and may be further exacerbated by an increasing prevalence of risk factors in many parts of the world [1].

Factors such as consuming a healthful diet, being physically active, avoiding tobacco use, and maintaining a healthful weight can have a strong influence on cancer prevention [2]. It has been estimated that approximately 50% of cancer cases can be prevented [3]. However, international cancer statistics continue to show that up to 80% of the cancer burden in high-income countries could be preventable in principle [4]. Therefore, there is considerable interest in studying the impact of lifestyle changes, in particular, the impact of changes in diet, on cancer development and progression [3]. There is compelling evidence that nutrition has substantial effects on the incidence and progression of cancer [5].

This narrative review summarizes the evidence for dried fruits and nuts and cancer incidence, mortality, and survival and their potential anticancer properties. In addition, the gaps that exist in the literature and recommendations for future research are discussed. The steps of study selection are described in Supplementary Figure S1.

## 2. Dietary Strategies to Prevent Cancer

Few countries have optimal diets for cancer prevention [6]. For example, analysis of diets by the Alternate Healthy Eating Index reveals suboptimal global levels due to either a relatively high consumption of red meat, added sugars, and transfat or a relatively low consumption of fruits, vegetables, nuts, and whole grains [7]. Suboptimal diets are estimated to cause 1.6 million preventable cancer deaths annually [7]. 

Nearly every authoritative health body recommends increased fruit intake for preventing cancer and chronic disease risk. The International Agency for Research on Cancer/World Health Organization (IARC/WHO) Report estimated that ~5% of cancer deaths in the U.S. are due to low fruit and vegetable consumption [6]. The U.S. Dietary Guidelines for Americans recommends 2 cups of fruit equivalents/day per 2000 kcal where ½ cup of dried fruit is 1 equivalent [8]. The U.S. Food and Drug Administration (FDA) also allows for a fruit and cancer claim provided the fruit meets the nutrient content requirements for a “good source” of at least vitamin A, vitamin C, or dietary fiber [9]. The model wording of the claim reads, “*Development of cancer depends on many factors. Eating a diet low in fat and high in fruits and vegetables, foods that are low in fat and may contain vitamin A, vitamin C, and dietary fiber, may reduce your risk of some cancers. Oranges, a food low in fat, are a good source of fiber and vitamin C*” [9]. The IARC/WHO European Code Against Cancer recommends the consumption of whole grains, pulses, vegetables, and fruits for cancer prevention [10].

Nuts, encompassing tree nuts and compositionally related peanuts, are also widely recommended for consumption as nutrient-dense foods [6,8,11]. The World Cancer Report associates dietary patterns containing high intakes of fruits and nuts with the reduction of colorectal cancer risk [6]. Several FDA-qualified claims about diet and cancer risk are relevant to the components found in dried fruit and nuts. Furthermore, other agencies have highlighted the importance of dietary bioactives in cancer prevention. The American Cancer Society guidance for consumers states, “Fruit and non-starchy vegetables contain a large number of potential anti-tumorigenic agents, such as dietary fiber, carotenoids” [11,12]. The World Cancer Research Fund International has published, “Fruit and non-starchy vegetables contain a large number of potential anti-tumorigenic agents”, among dietary guidance for cancer prevention [13].

## 3. Dried Fruits and Cancer

Many types of dried fruits are consumed worldwide. Globally, the most common dried fruits are raisins, dates, prunes, apricots, and figs [14]. However, other specialty dried fruits are produced, including sweetened-dried cranberry and high-value, freeze-dried fruits and powders. Globally, the Middle East and Europe account for half of dried fruit consumption [14]. In the U.S., dried fruit contributes <4% of total fruit intake, whereas juice and fresh or other nondried forms are more commonly consumed [15]. For this review, we mainly focus on cancer prevention through the most common dried fruits.

### 3.1. Preclinical Studies Relating Dried Fruits and Cancer

Cancer develops through many mechanisms. Preclinical studies have revealed that dried fruit may prevent cancer at the stage of initiation through the induction of detoxification enzymes and reducing the impact of carcinogens and environmental stress; during promotion by inhibiting oxidative stress and inflammation; and at the stage of progression by inducing apoptosis [16]. Mechanistic data from in vitro studies are available for raisins [17,18], apricots [19,20], figs [21,22], prunes [23,24], and dates [25,26] and in vivo for figs [27], dates [28], prunes [29], and apricots [30]. As reviewed elsewhere, these studies cover gastric, colon, breast, liver, bone, prostate, renal, and testicular cancers [16]. Furthermore, fruit bioactives have a direct impact on cancer-related factors, including antioxidant and anti-inflammatory activity and improved gut immune function in preclinical studies [16,31,32,33]. These functionalities may reduce cancer by inhibiting the risk of cancer initiation and progression and by improving survival.

Dried fruits contain fiber, micronutrients, and bioactives that may contribute to cancer chemoprevention, as summarized in a companion article [34]. Dried fruits contain a diverse number of bioactives, including phenolics, carotenoids, and terpenoids [16]. The drying process itself impacts the profile, abundance, and possibly the bioaccessibility of bioactives in dried fruits. For example, freeze-drying may retain bioactives more so than forced-air or sun-drying processes [35]. Heat can increase the formation of melanoidins, especially during the production of raisins [36]. Fruit melanoidins are poorly described but function as antioxidants and accumulate polyphenols into complex polymers. Thermal processing of dried fruits (and nuts) also increases the content of advanced glycation endproducts (AGEs) as compared to unprocessed fruits and nuts [37]. AGEs may play a role in carcinogenesis [38]. However, a large, multinational, prospective cohort study across 20 anatomical cancer sites reported that a higher intake of dietary AGEs was not associated with an increased risk of overall cancer and most cancer types studied. A nonlinear, weak positive association was observed between higher AGE intake and the risk of prostate cancer [38]. The addition of sulfites during processing inhibits melanoidin formation [39] and therefore may alter the profile of bioactives when used in the fruit drying process. 

Drying may also impact the structure and accessibility of soluble fibers and bioactives from fruit. In vitro digestion of different dried fruits may increase or decrease the release of antioxidants [40]. In mice, the consumption of nonextractable phenolics from dried berries increases colonic polyphenol content more so than the consumption of freely extractable polyphenols [41]. Therefore, drying may also impact the accessibility of fiber-associated phenolics and other bioactives that could subsequently affect the gut microbiota and intestinal immune system. Further studies are needed to link dried fruit processing parameters to cancer prevention activities.

### 3.2. Human Intervention Studies of Dried Fruits and Cancer

The evidence from human intervention studies for cancer prevention by dried fruit consumption is very limited. Thus far, human intervention studies with dried fruits have focused on cardiometabolic health or other chronic diseases [42,43,44]. Some evidence is available for the function of high-value, freeze-dried berry products in healthy individuals and those with colorectal, oral, and prostate cancers [45,46,47,48,49,50]. However, these forms of dried fruit are not commonly consumed and have a profile of bioactives similar to fresh fruits, which differs from more commonly available and consumed dried fruits produced via processes that utilize heat during drying. 

### 3.3. Epidemiological Studies of Dried Fruits and Cancer

#### 3.3.1. Cancer Incidence (or Risk)

Few epidemiological studies have directly assessed the relationship between dried fruits and cancer risk. A systematic review of observational studies published through 2018 found insufficient studies to perform a meta-analysis [51]. The review identified 16 studies with 12,732 cases from 437,298 participants that assessed cancer risk and the intake of total dried fruits or specifically raisins, prunes, dates, or figs [51]. Among the prospective studies, there was an inverse relation between total dried fruit consumption and cancer incidence in seven studies with a significant, dose-response trend identified in three studies on pancreatic cancer, prostate cancer, and colorectal polyps [51]. Among the case–control studies, there were inverse associations of cancer incidence with total dried fruits, raisins, or dates, with five of seven studies reporting significant associations [51]. Among these studies, dried fruit intake was associated with reduced stomach cancer, pancreatic cancer, and colorectal cancer incidence (for dates but not other dried fruits), nasopharyngeal cancer incidence (raisins but not dried figs, which increased risk), and bladder cancer incidence [51]. Where dried fruit intakes were compared with fresh fruit intakes, dried fruit was more protective than fresh fruit in five of seven prospective studies and three of four case–control studies [51]. 

Prospective cohort studies conducted in the Netherlands have shown no association between total dried fruit intake and urothelial [52], stomach [53], and prostate [54] cancer incidence. In contrast, consumption of prunes was associated with increased colorectal cancer risk in the Nurses’ Health Study and Health Professionals’ Follow-up Study (RR (95% CI) for women: 1.46 (0.93, 2.31) and for men: 1.73 (1.20, 2.50)) [55]. Several prospective analyses conducted in cohorts of Californian Seventh Day Adventists have shown that the frequency of dried fruit intake is associated with a lower risk of cancer [56,57,58]. In the Adventists Health Study 1, intake of raisins, dates, and other dried fruits ≥3 times/week was associated with a 65% reduction (relative risk (RR) (95% CI): 0.35 (0.17, 0.73)) in the relative risk of fatal pancreatic cancer compared to intake less than once per month [56]. In an analysis of the Adventists Health Study 1 and 2, dried fruit intake ≥3 times/week was associated with 24% lower odds of rectal/colon polyps (odds ratio (OR) (95% CI): 0.76 (0.58, 0.99)) compared to intake less than once per week [57]. Finally, intake of raisins, dates, or other dried fruits ≥5 times/week was associated with a lower relative risk of prostate cancer after adjustment for age ((RR (95% CI): 0.51 (0.31, 0.85)) compared to intake <1 time/week; however, the relationship was attenuated after further adjustment for education and other dietary factors (RR (95% CI): 0.62 (0.36, 1.06)) [58]. In an analysis of Adventist Health Study 1, no association was observed between dried fruit intake and lung cancer [59]. 

Recent epidemiological studies in other populations have investigated dried fruit intake and cancer risk (Table 1). 

The risk of breast, endometrial, and ovarian cancer was assessed in the prospective UK Women’s Cohort study of *n* = 35,372 women [60]. After 18 y of follow-up in participants aged 35–69, total dried fruit intake was associated with reduced risks of endometrial cancer (hazards ratio (HR) (95% CI): 0.60 (0.37, 0.97)) and postmenopausal endometrial cancer (HR (95% CI) 0.55: (0.31, 0.98)). Total dried fruit intake was not related to breast or ovarian cancer risks; total fruit intake was not associated with breast, endometrial, or ovarian cancer risks. 

Dried fruit intake and cancer risk were evaluated in the National Institutes of Health-American Association of Retired Persons Diet and Health study, which included 485,403 participants who were 50–71 years old [61]. In this study, dried fruit intake was associated with lower RR (95% CI) of both liver cancer (0.73 (0.60, 0.89)) and chronic liver disease mortality (0.59 (0.48, 0.73)) [61]. 

A two-sample Mendelian randomization study utilized ~500,000 samples from the UK Biobank database to investigate dried fruit intake and cancer risk [62]. In this data set, for one standard deviation increase in genetically predicted dried fruit intake, a reduced risk was observed for oral cavity/pharyngeal cancer, lung cancer, squamous cell lung cancer, breast cancer, pancreatic cancer, and cervical cancer [62].

Case–control analyses conducted in Australia, Jordan, Spain, and Turkey showed relationships between dried fruit intake and cancer incidence. An Australian case–control analysis reported that individuals with pancreatic cancer (cases) had a significantly lower intake of raisins than controls [63]. In a case–control study conducted in Jordan, a daily intake of dates (OR (95% CI): 0.52 (0.27, 0.98)) was associated with lower odds of colorectal cancer; total dried fruit intake was not associated with colorectal cancer risk [64]. In a case–control analysis conducted in Spain, a higher intake of dried fruits was associated with lower odds of gastric cancer (OR (95% CI): 0.40 (0.20, 0.80)) [65]. Likewise, in a Turkish case–control study, a higher intake of dried fruit was associated with a lower risk of gastric cancer [66]. 

#### 3.3.2. Cancer Mortality and Survival

Increased fruit intake is associated with reduced cancer mortality. A meta-analysis of 26 cohort studies reported that an intake of five servings of fruits and vegetables relative to the reference level of two servings had a hazard ratio (HR) (95% CI) of 0.90 (0.86, 0.95) for cancer mortality [67]. Few studies have directly analyzed dried fruits and cancer mortality. A meta-analysis published in 2017 identified only two studies on dried fruit and total cancer mortality risk and found a nonsignificant association (RR (95% CI): 0.89 (0.61, 1.30)) [68].

### 3.4. Research Gaps, Needs, and Priorities Related to Dried Fruits and Cancer

Although fruits are currently recommended for cancer prevention, more research is needed to understand the specific contributions and mechanisms of dried fruit intake and cancer prevention. Additional epidemiological studies are needed to assess dried fruit intake and additional cancer types. Dried fruit is often not treated as a separate subcategory of total fruit intake when assessing dietary intake, which hinders the ability to assess cancer risk and dried fruit intake in epidemiological studies [51]. Sufficient dried fruit intake in a study population should be considered. Although the intake on a g/day basis may be low, higher frequency intake of 3+ to 5+ times/week, such as in the AHS [57], may be considered reasonable intake frequencies to detect inverse associations between dried fruit consumption and cancer risk. At lower frequencies of intake, e.g., less than weekly, associations may be more difficult to detect. Furthermore, dried fruit intake is also difficult to describe since the composition and methods used to process dried fruit vary. Therefore, new assessment tools or biomarkers are needed to accurately assess intake. 

Additional preclinical and mechanistic studies are needed. However, greater attention to the design of nutritionally relevant studies is needed considering that many fruit bioactives are metabolized before reaching tissues and organs [69]. Identifying specific mechanisms by which dried fruit may impact cancer risk can inform the design of mechanistic intervention studies. Relating specific bioactives in dried fruits to preventive mechanisms is desirable. For example, the World Cancer Report links the intake of fruit carotenoids with a reduced risk of estrogen receptor-negative breast tumors [6,70]. Additional human studies are needed to understand how other bioactives in dried fruits affect cancer risk and survival. Assessing dried fruit bioactives and their metabolites in tissues and plasma in human studies can lead to new mechanistic insights and dietary recommendations. Lastly, future human intervention studies are needed to clarify the role of dried fruit in primary prevention, secondary prevention, or improving the quality of life in cancer patients. 

## 4. Tree Nuts, Peanuts, and Cancer

A holistic view of the evidence shows that most diets that are protective against cancer are rich in foods of plant origin [2]. Relatively unprocessed foods of plant origin are rich in nutrients and dietary fiber. Higher consumption of these foods instead of processed foods and sugars could protect against weight gain, overweightness, and obesity [71] and therefore protect against obesity-related cancers, such as postmenopausal breast, colorectum, liver, thyroid, pancreas, endometrium, and kidney cancer [2]. A higher consumption of nuts has been associated with less weight gain in adults as summarized in a systematic review and meta-analysis of prospective cohorts and randomized controlled trials [72]. Nuts, including tree nuts (almonds, hazelnuts, and walnuts) and peanuts, contain high amounts of nutrients such as unsaturated fats, protein, vitamins (e.g., α-tocopherol, folate, and niacin), nonsodium minerals (e.g., magnesium, calcium, and potassium), and phytochemicals—all of which may have anticarcinogenic, anti-inflammatory, and antioxidant properties. Nuts, and walnuts, in particular, have also been shown to modulate the microbiota by increasing gut microbial diversity [73], and new mechanistic hypotheses on diet and cancer relationships include interactions between host and environmental factors in selecting the microbiota that in turn influence carcinogenesis [74].

### 4.1. Preclinical Studies Related to Nuts and Cancer

In vitro and in vivo studies to determine whether nuts can help combat cancer are instrumental to understanding potential mechanisms and whether results are coherent with studies in humans. 

Preclinical studies for breast cancer have demonstrated reduced growth and multiplicity of breast cancer tumors in relation to walnuts or the main compounds characteristic of walnuts, such as melatonin [75,76]. Potential mechanisms for cancer prevention include the suppression of proliferation, alterations in cell signaling pathways involved in cell differentiation and apoptosis, and selective inhibition of some cyclooxygenase and lipoxygenase activities [75,76,77].

Preclinical studies for colon cancer suggest that consumption of mixed nuts, walnuts, and almonds inhibits DNA damage and tumor growth through the suppression of angiogenesis, proliferation, and inflammation, as well as increased apoptosis and favorable alterations to the gut bacteria and enterotype-like clusters [78,79,80,81,82,83]. In line with the latter, a cross-sectional study among 222 Koreans showed that a healthy dietary pattern characterized by higher intakes of nuts/seeds was related to higher α-diversity reflecting gut microbial health [84].

In vivo and in vitro models for prostate cancer suggest that diets containing walnuts and almonds may reduce the risk of prostate cancer through declines in plasma levels of IGF-1, resistin, LDL-cholesterol, oxidative stress, and inflammation, and increased expression of tumor suppressors [85,86,87].

Bioactives in nuts with anticancerogenic potential that have been studied in preclinical studies include lipid-associated components [82], such as ellagic acid, which is a dietary flavonoid polyphenol abundant in walnuts and pecans [80], and melatonin found in walnuts along with polyunsaturated fatty acids, which are abundant in nuts in general [76]. Another bioactive nutrient in nuts is selenium, for which Brazil nuts are one of the richest known food sources [88]. In a 2023 preclinical study in mice, selenium-rich Brazil nuts and selenomethionine dietary supplementation reduced mammary tumor growth [89]. Another aspect to highlight is that differences in the composition of bioactives across various types of nuts likely translate into different potential anticancer properties.

These potential anticancer properties of nuts based on preclinical studies are summarized in Table 2.

### 4.2. Human Intervention Studies of Nuts and Cancer

Several nuts have been evaluated in human intervention studies for cancer-relevant outcomes. These studies have illustrated that both long- (>2 months) and shorter-term (≤8 weeks) consumption of nuts may modulate biochemical pathways relevant to cancer prevention and reduce cancer progression.

In a randomized, controlled trial involving 4282 women aged 60 to 80 years at high cardiovascular disease risk, a Mediterranean diet supplemented with mixed nuts (30 g/d: 15 g walnuts, 7.5 g hazelnuts, and 7.5 g almonds) vs. the advice to follow a low-fat diet was associated with a risk reduction of first invasive breast cancer with a hazard ratio of 0.59 (95% CI: 0.26 to 1.35) [91]. However, this association was not significant, most likely due to a small number of incident cases (*n* = 35) during the relatively short follow-up of 4.8 years [91]. 

In a randomized clinical trial involving 32 participants (>50 years of age), Hu et al. found that a 6-week intervention with Brazil nuts (6 nuts/day) or green tea extract alone affected gene expressions associated with selenoproteins, WNT signaling, inflammation, and DNA methylation comparing baseline to end levels after 6 weeks [88], all of which are genetic and epigenetic biomarkers related to colorectal cancer development. Brazil nuts are also an excellent source of selenium, and there is evidence from observational studies in humans that selenium intake/status may play a protective role in colorectal cancer development in European populations where selenium status is lower as compared to the USA [92]. However, a large prevention trial (SELECT) in North America failed to show any reduction in cancer incidence, cancer mortality overall, or for specific cancers, including prostate, lung, or colorectal cancer with selenium supplementation [93].

In a randomized crossover study in 40 middle-aged men, no significant difference between mean prostate-specific antigen (PSA) levels at the conclusion of the 6-month walnut-supplemented diet phase (1.05 mu g/L, 95% CI [0.81, 1.37]) and the conclusion of the 6-month Western-type control diet phase (1.06 mu g/L, 95% CI [0.81, 1.38]) (*p* = 0.86) was observed [94].

In contrast, in an 8-week walnut supplementation time course experiment to examine the effects of walnuts on serum tocopherols (T) and PSA in 21 men, a significant decrease in the α-T: γ-T ratio with an increase in serum γ-T and a trend towards an increase in the ratio of free PSA: total PSA was observed, which suggests that walnuts may improve biomarkers of prostate and vascular status [95].

Jia et al. investigated the effects of almond consumption on DNA damage and oxidative stress among thirty regular cigarette smokers randomly divided into three groups [96]. After four weeks, lower levels of urinary 8-hydroxy-2′-deoxyguanosine (8-OH-dG) and single-strand DNA breaks and lower malondialdehyde levels, a surrogate oxidative stress marker, were observed in the two almond-treated groups compared with the control group that did not receive any almonds [75].

In addition, emerging evidence based on randomized, controlled trials suggests that nut consumption has a favorable impact on the gut microbiota [73]. A meta-analysis of randomized controlled trials including 9 trials (almonds, *n* = 5; walnuts *n* = 3; and pistachios, *n* = 1) showed that nut consumption significantly increased the relative abundances of butyrate-producing bacteria, such as *Clostridium*, *Lachnospira*, and *Roseburia*, that have been associated with the prevention of cardiometabolic diseases and certain cancers, such as colorectal cancer. For example, in a randomized, controlled crossover study in healthy Caucasian adults (*n* = 194), 8 weeks of walnut consumption (43 g/day) compared to a nut-free diet significantly enhanced gut microbial diversity [97].

### 4.3. Epidemiological Studies of Nuts and Cancer

#### 4.3.1. Cancer Incidence

In a 2021 systematic review and dose-response meta-analysis, a higher intake of total nuts (per 5 g/day increment) was associated with a lower incidence of cancers of the colon, lung, pancreas, and breast with relative risks equal to 0.75 (95% CI 0.60–0.94), 0.97 (95% CI 0.95–0.98), 0.94 (95% CI 0.89–0.99), and 0.98 (95% CI 0.96–0.99), respectively [98]. A lower incidence of several other types of cancer was also observed comparing the highest vs. lowest intake of total nuts, including cancers of the esophagus, stomach, rectum, liver, ovaries, endometrium, and leukemia; however, the relative risk estimates were imprecise and included the null; hence, no firm conclusions for these cancers can be drawn yet. In addition, for some cancer sites (e.g., colon, breast), results from case–control studies (which are more likely to be affected by recall and selection biases than cohort studies) were combined with those from cohort studies in this meta-analysis [98]; thus, additional prospective cohort studies are needed. These findings are largely similar to a 2020 meta-analysis of prospective studies with the exception of breast cancer where no association with intake of total nuts was observed [99]. A 2023 meta-analysis that included only prospective studies reported an inverse association between total nut intake (highest vs. lowest) and the risk of cancers of the lung and stomach with pooled relative risks equal to 0.86 (CI: 0.81–0.91) and 0.79 (95% CI: 0.68–0.91), respectively [100]. The few cohort studies that investigated the association between nut consumption and total cancer incidence in cohorts from Europe and the USA with more than 20 years of follow-up and more than a total of 60,000 incident cancer events reported no clear association of 5+ times per week vs. never/or almost never consuming a serving of nuts [101,102,103]. 

#### 4.3.2. Cancer Mortality and Survival

**Figure 1:**
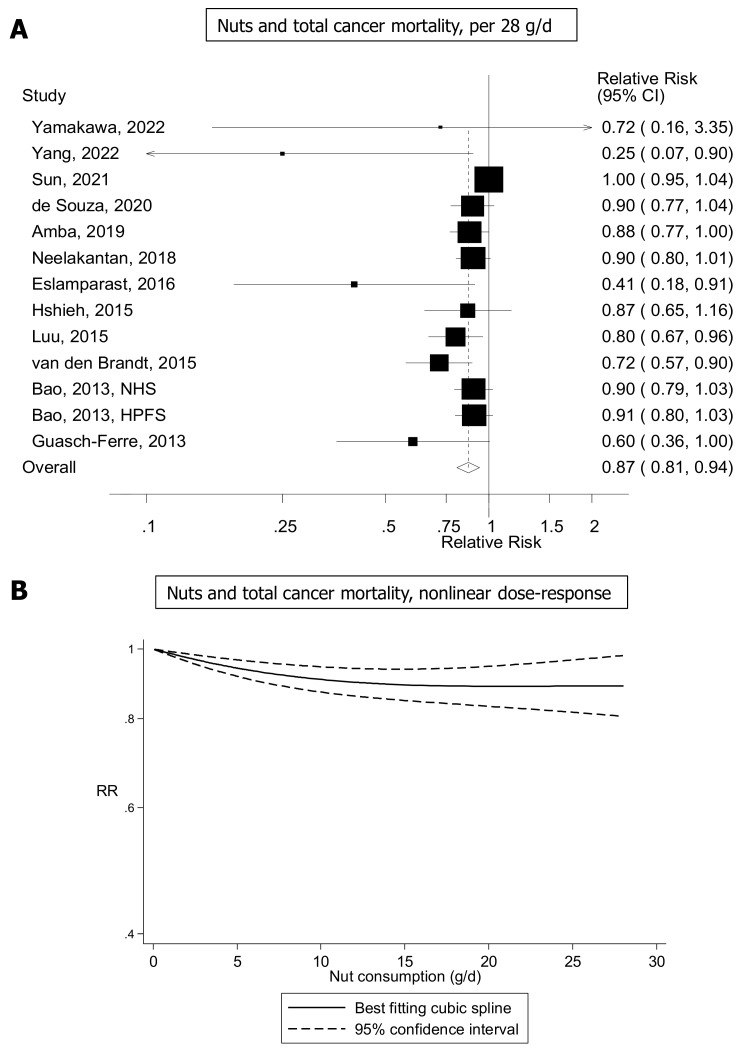
Nuts and total cancer mortality, linear (**A**), and nonlinear (**B**) dose-response analysis (updated analysis based on Aune et al.) [106,110,111,112,113,114,119,120,121,122,123,124].

In an umbrella review of epidemiological evidence that included 49,161 cancer deaths, a daily intake of 28 g/d nuts was inversely associated with cancer mortality with an RR of 0.89 (95% CI: 0.83–0.94) [104]. The proportion of variability due to between-study heterogeneity was low (I^2^: 23%), and the strength of the evidence was rated as moderate (AMSTAR-2) [104]. The authors reported slightly stronger inverse associations with cancer mortality for tree nuts than for peanuts [104]. These findings are in line with previous systematic reviews and meta-analyses of prospective studies [100,105,106,107,108]. For example, Chen et al. [105] estimated a summary RRs for high (5+ servings/week) compared with low nut (never/almost never) consumption of 0.87 (95% CI: 0.80–0.93) for cancer mortality (11 studies 21,353 deaths), while Aune et al. reported a summary estimate of 0.83 (95% CI: 0.75–0.92) per 28 g/d for cancer mortality [106]. When updating the latter analysis by adding results from 6 additional cohort studies that have since been published [109,110,111,112,113,114] (Supplementary Table S1), the summary estimate was 0.87 (95% CI: 0.81–0.94, I^2^ = 60%, *n* = 13) per 28 g/d (Figure 1A) based on data from 13 cohort studies (12 publications), and there was evidence of nonlinearity (*P*_nonlinearity_ = 0.005) with most of the reductions in risk observed with an intake of 15–20 g/day (Figure 1B). 

There is also evidence that among patients with cancer, frequent consumption of nuts may be linked to lower mortality from all causes. In the Health Professionals Follow-up Study, frequent nut intake (5 or more times per week; serving size: 28 g) among patients with prostate cancer was associated with a lower risk of dying from prostate cancer and from all causes by more than 30 percent compared to the men who ate nuts once or less a month [115]. 

In a prospective study among 3449 long-term breast cancer survivors with 374 deaths, including 252 breast cancer deaths, total nut intake ≥ 17 g/week compared to nonconsumption was inversely associated with overall survival (OS) (HR (95% CI): 0.74 (0.52, 1.05)) and disease-free survival (DFS) (0.48 (0.31, 0.73)), and these associations did not vary by nut type [116].

Among 826 patients with stage III colon cancer in a prospective, observational study, compared to nonconsumers, patients who consumed ≥ 2 servings of nuts (1 oz per serving) per week had an HR (95% CI) of 0.43 (0.25, 0.74) for OS, of 0.58 (0.37, 0.92) for DFS, and 0.70 (0.42, 1.16) for recurrence-free survival (RFS) compared to nonconsumers [117]. When cumulative averages of nut consumption before and after diagnosis were used for the statistical analysis, the corresponding HRs (95% CIs) were 0.43, (0.30–0.61), 0.45 (0.33–0.62), and 0.46 (0.32–0.64), respectively, suggesting the importance of repeated dietary assessments [110]. Subgroup analyses showed that the beneficial effects of nut intake were particularly attributable to tree nut intake [117]. Similarly, in a prospective, observational study among 1404 long-term colorectal cancer survivors, compared to the lowest nut intake (1st quintile), the highest nut intake (5th quintile) postdiagnosis was inversely associated with OS (HR (95% CI): 0.48 (0.31, 0.75) [118].

### 4.4. Research Gaps, Needs, and Priorities Related to the Study of Nuts and Cancer

Considering the current evidence of the relationship of nut consumption with cancer risk and mortality where nut consumption is often only assessed at baseline, and potential changes in consumption patterns over time are missed, more research with improved exposure assessment is warranted. This should include repeated assessment of the intake of specific types of nuts and improved quantification. Better biomarkers of nut consumption would also help to further investigate the promising observations of the putative chemopreventive effect of nuts in cancer development, including secondary and primary cancer outcomes. Furthermore, there is a need to investigate additional cancer types, including thyroid, kidney, or head and neck, and outcome-wide analyses across cancer sites could provide answers relatively quickly. The multifaceted nature of cancer risk and the variety of cancer chemopreventive mechanisms suggest that nuts may have different effects among cancer subtypes. To address the heterogeneity across studies and investigate rare types of cancer, a pooling of prospective cohorts could be a way forward.

For cancer prognosis, additional studies with pre- and postdiagnosis dietary assessment are warranted. It is likely that distinguishing the timing of nut consumption could provide a greater understanding of how nuts may modify risks during different stages of cancer development. 

Few studies have addressed the impact of nuts on the co-occurrence of cancer and other long-term chronic diseases (e.g., diabetes) in an individual. For example, in a multinational cohort study, we showed that greater adherence to the Mediterranean diet was inversely associated with cardiometabolic multimorbidity after a first primary cancer [125].

## 5. Summary and Recommendations

The degree of evidence for dried fruit consumption and cancer prevention is more limited than what has been established for nuts. Preliminary studies have been promising and have begun to establish mechanistic links between bioactives and chemopreventive pathways. Commonly consumed dried fruits have different profiles of bioactives than their fresh counterparts. Several studies suggest that dried fruit intake is associated with a greater reduction of risk than that of fresh fruit. As with nuts, improved methods to track intake and document consumption across multiple types of dried fruit are needed to improve epidemiological studies for diet and cancer prevention. 

Evidence from multiple lines of research encompassing cell line studies, animal models, observational studies, interventional studies, and meta-analyses is suggestive that a higher consumption of nuts is inversely associated with the risk of certain cancers and of dying from cancer. Among the 12 cancer sites investigated in the literature (i.e., esophagus, stomach, colorectum, liver, pancreas, lung, breast, ovary, endometrium, leukemia, prostate, and lymphomas), inverse associations were most consistent across studies for the incidence of colorectal cancer and, more specifically, with colon cancer. Studies differentiating between tree nuts and peanuts tend to report stronger inverse associations between tree nuts and cancer incidence and cancer mortality than for peanuts. There is limited, inconclusive evidence for an inverse association with the incidence of cancers of the pancreas, stomach, and lungs, while the most recent studies on breast cancer are null. Evidence is largely missing for other types of cancer; however, there is consistent evidence that nut consumption is associated with reduced total cancer mortality. Potential mechanisms include suppression of angiogenesis, proliferation, and inflammation, as well as increased apoptosis and favorable induced changes in gut bacteria. Indirectly, a higher consumption of nuts may be linked to a reduced risk of certain cancers or dying from cancer through reduced weight gain during adult life. Nuts in the diet may also have a role in tertiary prevention in cancer survivors where a higher nut intake was consistently related to better survival of cancers of the colorectum, breast, and prostate. Considering the observation that nut consumption appears to be more consistently associated with reduced total cancer mortality than total cancer incidence, it is also possible that an association with cancer is driven more so by improvements in survival after a cancer diagnosis than reductions in cancer incidence, but further studies are needed to clarify this, as the number of studies on total cancer incidence is limited. 

## Figures and Tables

**Table 1:** Epidemiological studies assessing the association between dried fruit intake and cancer risk published since 2019.

Study Type	Participants	Cancer Type	Outcome (95% CI)	Reference
Systematic review	*n* = 437,298 from 16 studies	Pancreatic, prostate, colorectal polyps	Dose-response trend from prospective studies	Mossine et al., 2020 [51]
		Stomach, pancreatic, colorectal, nasopharyngeal, bladder	Total dried fruit, raisins, or dates reduced incidence from case–control studies	
Cohort	UK Women’s Cohort Study (*n* = 35,372 women aged 35–69 in England, Wales, and Scotland)	Breast	HR 1.04 (0.98,1.13)	Dunneram et al., 2019 [60]
		Endometrial	HR 0.60 (0.37, 0.97)	
		Ovarian	HR 1.06 (0.89, 1.26)	
Prospective cohort	National Institutes of Health-American Association of Retired Persons Diet and Health Study (*n* = 485,403 men and women aged 50–71 at baseline in the United States)	Liver	HR (Q5 vs. Q1) 0.73 (0.60, 0.89)	Zhao et al., 2022 [61]
Mendelian randomization	UK Biobank (*n* ~500,000 men and women aged 49–69 in the United Kingdom)	Oral cavity/pharyngeal	IVW OR 0.17 (0.04, 0.69)	Jin et al., 2022 [62]
		Lung	IVW OR 0.33 (0.17, 0.64)	
		Squamous cell lung	IVW OR 0.23 (0.09, 0.60)	
		Breast	IVW OR 0.47 (0.32, 0.68)	
		Pancreatic	IVW OR 0.03 (0.001, 0.68)	
		Cervical	IVW OR 0.99 (0.9897, 0.9998)	
		Lung adenocarcinoma, endometrial, thyroid, prostate, bladder, brain	IVW OR not significant	

Abbreviations: HR, Hazard ratio; IVW, Inverse variance weighted; OR, Odds ratio.

**Table 2:** Potential anticancer properties of nuts based on preclinical studies.

Author Year	Cancer Model	Putative Mechanism of Nuts Dietary Factor	Dietary Factor
Breast Cancer-Related Studies			
Hardman and Ion 2008 [90]	Human breast cancer tumors in nude mice	Suppression of cell proliferation or suppression of metastasis	18% of dietary calories from walnuts
Hardman et al., 2011 [75]	C(3)1 TAg transgenic mice, breast cancer	Alterations in cell signaling related to proliferation, differentiation, and apoptosis	Walnuts in the diet
Garcia et al., 2015 [76]	Implanted mammary gland adenocarcinoma in BALB/c mouse model	Inhibition of cyclooxygenase and lipoxygenase	6% walnut oil or 6% walnut flour containing phytomelatonin
Chen et al., 2015 [77]	Breast cancer cells	Growth inhibition of breast cancer cells through cell cycle arrest and inhibition of proliferation	Ellagic acid that is abundant in walnuts
Colorectal Cancer-Related Studies			
Hong et al., 2022 [78]	Colonic cell proliferation, apoptosis, and gene expression in rat model	Reduced DNA damage possibly via downregulation of RelA inflammation gene expression without changes to colonic cell proliferation and apoptosis	Mixed nuts in the diet
Chen et al., 2020 [79]	Mouse tumor bioassay after colonotropic carcinogen exposure	Favorably altering the gut microbiota	Walnuts in a Western diet
Nagel et al., 2012 [80]	HT-29 human colon cancer cells in nude mice	Inhibition of tumor growth rate through suppression of angiogenesis	Walnut and flaxseed oil
Nakanishi et al., 2016 [81]	Mice treated with organotropic colon carcinogen	Tumor suppression associated with alterations in gut bacteria	Dietary walnut of up to 15% of total caloric intake
Davis and Iwahashi 2001 [82]	Aberrant crypt foci (ACF) in rats treated with azoxymethane	ACF and cell turn over reduced	Whole almond-, almond meal- or almond oil-containing diet
Prostate Cancer-Related Studies			
Davis et al., 2012 [85]	Transgenic adenocarcinoma of the mouse prostate (TRAMP)	Reduced TRAMP mouse prostate cancer growth and size; declines in plasma IGF-1, resistin, and LDL	Whole almonds as part of a high-fat diet
Kim et al., 2014 [86]	TRAMP	Reduced TRAMP mouse prostate cancer growth and size; improved insulin sensitivity and effects on cellular energy status, tumor suppression	Whole walnuts, walnut oil
Reiter et al., 2013 [87]	Implanted tumor model in nude mice	Reduced number and growth of LNCaP human prostate cancer cells; decreased oxidative stress	Standard mouse diet supplemented with walnuts

## Data Availability

Not applicable.

## Supplementary Materials

The following supporting information can be downloaded at: https://www.mdpi.com/article/10.3390/nu15061443/s1, Figure S1: PRISMA Flow Diagram of Study Selection; Table S1: Prospective cohort studies on total nut consumption and total cancer mortality. References [126,127] are cited in the supplementary materials.
