# Supplementary material for: Dried Fruits, Nuts, and Cancer Risk and Survival: A Review of the Evidence and Future Research Directions
Source: Nutrients. 2023 Mar 16;15(6):1443. doi: 10.3390/nu15061443 (PMC10051070; doi:10.3390/nu15061443)
Supplement: Supplementary file 1 [file nutrients-15-01443-s001.zip › Supplementary Figure S1. PRISMA flow diagram_Nuts_DriedFruits_Cancer_HN.pdf]

Supplemental Figure S1: PRISMA 2009 Flow Diagram

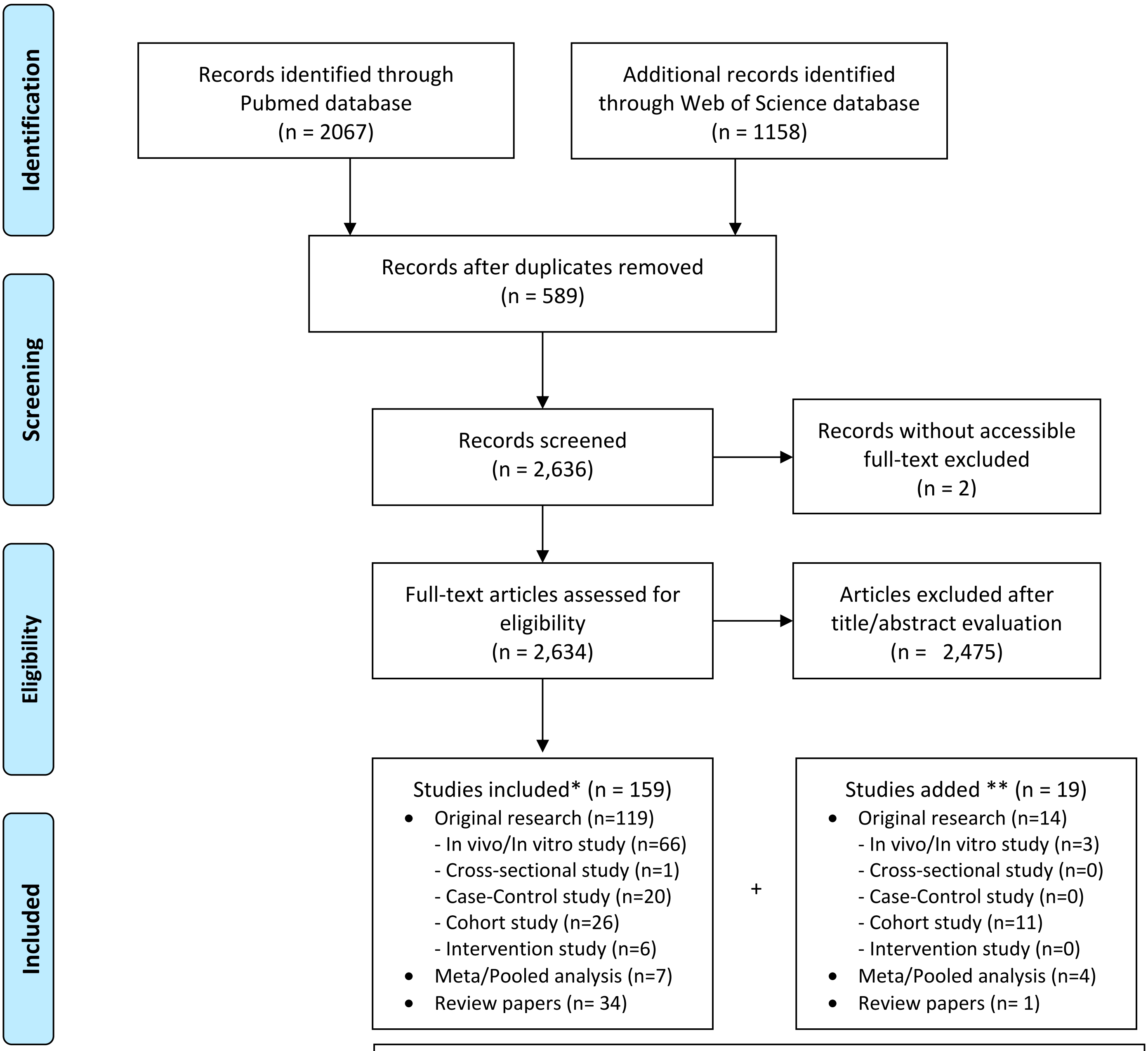

* Freisling, H.; Noh, H. Nut Consumption and Cancer. In *Health Benefits of Nuts and Dried Fruits*, Cesarettin Alasalvar, J.S.-S., Emilio Ros, Joan Sabate, Ed.; CRC Press: 2020; pp 1-20, DOI: 10.1201/9781315173337.
** Bolling, B. et al. Dried Fruits, Nuts, and Cancer Risk and Survival: A Review of the Evidence and Future Research Directions. Nutrients

From: Moher D, Liberati A, Tetzlaff J, Altman DG, The PRISMA Group (2009). *P*referred *R*eporting *I*tems for *S*ystematic Reviews and *M*eta-*A*nalyses: The PRISMA Statement. PLoS Med 6(7): e1000097. doi:10.1371/journal.pmed1000097

For more information, visit www.prisma-statement.org.
