# Supplementary material for: Dried Fruits, Nuts, and Cancer Risk and Survival: A Review of the Evidence and Future Research Directions
Source: Nutrients. 2023 Mar 16;15(6):1443. doi: 10.3390/nu15061443 (PMC10051070; doi:10.3390/nu15061443)
Supplement: Supplementary file 1 [file nutrients-15-01443-s001.zip › Supplementary Table S1. Total nut consumption and total cancer mortality.pdf]

Supplementary Table S1. Prospective cohort studies on total nut consumption and total cancer mortality

| Author, publication year, country | Study name | Study period | Number of participants, gender, age, number of deaths | Dietary assessment | Exposure and subgroup | Nut consumption frequency or amount | Relative risks (95% confidence intervals) | Adjustment for confounding factors |
|---|---|---|---|---|---|---|---|---|
| Bao Y et al, 2013, USA | Nurses' Health Study | 1980-2010, 30 years follow-up | 76464 women, age 34-59 years: 6535 cancer deaths | Validated FFQ, 61-116 food items | Nuts | Never<br><1/wk<br>1<br>2-4<br>≥5 | 1.00<br>0.94 (0.88-1.01)<br>0.91 (0.84-0.99)<br>0.89 (0.82-0.98)<br>0.94 (0.82-1.09) | Age, race, BMI, physical activity, smoking, screening, multivitamin use, aspirin use, FH – DM, MI or cancer, history of DM, hypertension, or hypercholesterolemia, total energy, alcohol, red or processed meat, fruits, vegetables, menopausal status, HRT |
| Bao Y et al, 2013, USA | Health Professional's Follow-up Study | 1986-2010, 24 years follow-up | 42498 men, age 45-70 years: 3758 cancer deaths | Validated FFQ, 131 food items | Nuts | Never<br><1/wk<br>1<br>2-4<br>≥5 | 1.00<br>0.91 (0.82-1.01)<br>0.98 (0.88-1.09)<br>0.95 (0.85-1.05)<br>0.86 (0.75-0.98) | Age, race, BMI, physical activity, smoking, screening, multivitamin use, aspirin use, FH – DM, MI or cancer, history of DM, hypertension, or hypercholesterolemia, total energy, alcohol, red or processed meat, fruits, vegetables |
| Guasch-Ferre M et al, 2013, Spain | PREDIMED Study | NA-NA, 4.8 years follow-up | 7216 men and women, age 55-80 years: 130 cancer deaths | Validated FFQ, 137 food items | Nuts | Never<br>1-3 serv/wk<br>>3 | 1.00<br>0.79 (0.52-1.20)<br>0.60 (0.37-0.98) | Age, sex, and intervention group, BMI, smoking status, education, leisure time physical activity, DM, hypercholesterolemia, oral antidiabetic medication, antihypertensive medication, use of statins, total energy, vegetables, fruits, red meat, eggs, fish, alcohol, Mediterranean diet adherence |
| Hshieh TT et al, 2015, USA | Physicians' Health Study | 1999-2002 – NA, 9.6 years follow-up | 20742 men, mean age 66 years: 868 cancer deaths | FFQ, 19 food items (validated in other cohorts) | Nuts | <1 serv/mo<br>1-3<br>1 serv/wk<br>2-4<br>≥5 | 1.00<br>0.91 (0.77-1.08)<br>0.88 (0.72-1.07)<br>0.87 (0.68-1.09)<br>0.87 (0.66-1.15) | Age, BMI, alcohol, smoking, exercise, calories, SFA, fruit and vegetables, red meat, prevalent DM, hypertension |

| | | | | | | | | |
|---|---|---|---|---|---|---|---|---|
| Luu HN et al, 2015, USA | Southern Community Cohort Study | 2002-2009 - NA, 5.4 years follow-up | 71764 men and women, age 40-79 years: 1551 cancer deaths | FFQ, 89 food items | Total nuts and peanut butter, African Americans<br><br><br><br><br>Total nuts and peanut butter, Caucasians | <0.95 g/d<br>0.95-<3.08<br>3.08-<7.30<br>7.30-<18.45<br>≥18.45<br><0.95 g/d<br>0.95-<3.08<br>3.08-<7.30<br>7.30-<18.45<br>≥18.45 | 1.00<br>0.89 (0.73-1.07)<br>0.85 (0.69-1.05)<br>0.91 (0.74-1.11)<br>0.74 (0.60-0.92)<br>1.00<br>0.98 (0.72-1.32)<br>0.89 (0.65-1.21)<br>0.68 (0.48-0.94)<br>0.93 (0.68-1.29) | Age, sex, race, education, occupation, household income, marital status, smoking pack-years, alcohol, BMI, physical activity, vitamin supplement use, Charlson Comorbidity Index, metabolic conditions (hypertension, heart disease, DM, obesity, hypercholesterolemia), total energy, red meat, chicken and duck intake, seafood, vegetables, fruits |
| van den Brandt PA et al, 2015, Netherlands | Netherlands Cohort Study | 1986-1996, 10 years follow-up | 3202 subcohort members, men and women, age 55-69 years: 3917 cancer deaths | Validated FFQ, 150 food items | Total nuts | 0 g/d<br>0.1-<5<br>5-<10<br>≥10 | 1.00<br>0.92 (0.81-1.05)<br>0.82 (0.68-0.98)<br>0.79 (0.67-0.93) | Age, sex, cigarette smoking, number of cigarettes per day, years of smoking, hypertension, DM, body height, BMI, non-occupational physical activity, highest level of education, alcohol, vegetables, fruits, energy, nutritional supplement use, women: HRT |
| Eslamparast T et al, 2016, Iran | Golestan Cohort Study | 2004 - 2013, 7 years follow-up | 50045 men and women, age ≥40 years: 887 cancer deaths | Validated FFQ | Nuts, all<br><br><br><br>Nuts, women<br><br><br><br>Nuts, men | Never<br><1 serv/wk<br>1-<3<br>≥3<br>Never<br><1 serv/wk<br>1-<3<br>≥3<br>Never<br><1 serv/wk<br>1-<3<br>≥3 | 1.00<br>0.96 (0.82-1.11)<br>0.84 (0.65-1.07)<br>0.62 (0.38-1.01)<br>1.00<br>0.91 (0.73-1.14)<br>0.72 (0.48-1.07)<br>0.43 (0.18-1.01)<br>1.00<br>0.98 (0.80-1.20)<br>0.90 (0.65-1.25)<br>0.73 (0.41-1.33) | Age, sex, BMI, education, place of residence, smoking status, opium, alcohol, physical activity, wealth score, diabetes, hypertension, total energy, fish, red meat, chicken, fruits, vegetables, dairy products, eggs, total fiber, magnesium, zinc, copper |
| Neelakantan N et al, 2018, Singapore | Singapore Chinese Health Study | 1993-1998 - 2014, 17.2 years follow-up | 57078 men and women, age 45-74 years: 5306 cancer deaths | Validated FFQ, 165 food items | Nuts | Per 28 g/d (nuts) or 16 g/d (peanut butter) | 0.92 (0.84-1.01) | Age, sex, total energy, dialect, education, smoking status, cigarettes per day, physical activity, sleep duration, BMI, diabetes mellitus, hypertension, vegetables, fruit, whole grains, nuts, legumes, dairy products, fish, sugar-sweetened beverages and fruit juice, alcohol |

| | | | | | | | | |
|---|---|---|---|---|---|---|---|---|
| Amba V et al, 2019, USA | NIH-AARP Diet and Health Study | 1995-1996 - NA, 15.5 years follow-up | 374101/380351 men and women, age 50-71 years: 26685 cancer deaths | Validated FFQ, 124 food items | Nuts | 0 g/1000 kcal/d<br>0.11<br>0.51<br>2.20<br>Per 2.15 g/d | 1.00<br>0.91 (0.87-0.95)<br>0.88 (0.83-0.92)<br>0.88 (0.84-0.92)<br>0.99 (0.98-1.00) | Age, sex, BMI, education, race, self-reported health condition, smoking status, cigarettes per day, total energy, alcohol, vitamin use, physical activity, white meat, red meat, whole grains, vegetables, fruits |
| de Souza RJ et al, 2020, International | The Prospective Urban Rural Epidemiology (PURE) study | 2003-2019 - 2019, 9.5 years follow-up | 124329 men and women, age 35-70 years: 1768 cancer deaths | Validated FFQ | Nuts | <30 g/mo<br>30 g/mo-<30g/wk<br>30 g/wk-<120 g/wk<br>≥120 g/wk<br>Per 30 g/d | 1.00<br>0.91 (0.79-1.06)<br>0.95 (0.82-1.10)<br>0.81 (0.65-1.00)<br>0.89 (0.76-1.04) | Age, sex, follow-up time, urban/rural location, center, lifestyle factors, education, tobacco use, BMI, WHR, physical activity, family history of CVD, DM and cancer, fish, fruits, vegetables, red/processed meat, legumes, total energy |
| Sun Y et al, 2021, USA | Women's Health Initiative | 1993-1998-2017, 18.3 years follow-up | 102521 women, age 50-79 years: 7516 cancer deaths | Validated FFQ, 122 food items | Nuts | 1<br>2<br>3<br>4<br>5<br>Per oz equivalent/day | 1.00<br>0.95 (0.88-1.02)<br>0.94 (0.87-1.01)<br>0.95 (0.89-1.03)<br>0.99 (0.92-1.06)<br>1.00 (0.95-1.04) | Age, race/ethnicity, education, income, Observational Study/Clinical Trials, unopposed estrogen use, estrogen+progesterone use, smoking status, physical activity, alcohol intake, total energy intake, DM, high blood cholesterol, FH - heart attack/stroke, whole grains, vegetables, fruits, sugar-sweetened beverages, unprocessed red meat, processed red meat, poultry, fish/shellfish, eggs, dairy products, legumes |
| Yang J et al, 2022, Hong Kong | Mr. Osteoporosis and Ms. OS study | 2001-2003 - 2017, 13.75 years follow-up | 3995 men and women, age ≥65 years: 469 cancer deaths | Validated FFQ, 280 food items | Nuts, all<br><br><br><br>Nuts, men<br><br><br><br>Nuts, women | <0.21 g/d<br>0.21-1.63<br>1.63-4.23<br>>4.23<br><0.41 g/d<br>0.41-1.98<br>1.98-5.12<br>>5.12<br>0.00 g/d<br>0.00-1.15<br>1.15-3.54<br>>3.54 | 1.00<br>0.84 (0.65-1.08)<br>0.87 (0.68-1.12)<br>0.72 (0.55-0.94)<br>1.00<br>0.87 (0.62-1.21)<br>0.93 (0.67-1.28)<br>0.67 (0.48-0.94)<br>1.00<br>0.81 (0.54-1.20)<br>0.79 (0.52-1.19)<br>0.84 (0.54-1.33) | Age, sex, energy intake, BMI, physical activity, systolic blood pressure, medical history - DM, hypertension, stroke, heart attack, angina, congestive heart failure, cancer, smoking habits, alcohol, education |

| | | | | | | | | |
|---|---|---|---|---|---|---|---|---|
| Yamakawa et al, 2022 | The Takayama Study | 1992-2008, 14.1 years follow-up | 31552 men and women, age ≥35 years: 1620 cancer deaths | Validated FFQ, 169 food items | Total nuts, men<br><br><br><br>Total nuts, women | 0 g/d<br>0.8<br>1.5<br>3.0<br>0.1 g/d<br>0.9<br>1.4<br>2.6 | 1.00<br>0.87 (0.71-1.06)<br>0.85 (0.69-1.06)<br>0.84 (0.68-1.03)<br>1.00<br>1.01 (0.78-1.29)<br>0.96 (0.74-1.26)<br>1.18 (0.93-1.51) | Age, marital status, education, BMI, diabetes, hypertension, smoking status, alcohol intake, physical activity, use of any vitamin supplement, dietary intake of total energy, vegetables, fruits, and red meat, and for women only: menopausal status |

BMI=body mass index, CVD=cardiovascular disease, DM=diabetes mellitus, FFQ=food frequency questionnaire, FH=family history, HRT=hormone replacement therapy, MI=myocardial infarction, NA=not available, SFA=saturated fatty acids, WHR=waist-to-hip ratio
